# Coring Revisited: A Case Report and Literature Review

**DOI:** 10.7759/cureus.29750

**Published:** 2022-09-29

**Authors:** Justin L Hruska, Wael Saasouh, Muhammad S Alhamda

**Affiliations:** 1 Anesthesiology, Detroit Medical Center, Detroit, USA; 2 Research, NorthStar Anesthesia, Detroit, USA; 3 Outcomes Research Consortium, Cleveland Clinic, Cleveland, USA

**Keywords:** prefilled syringe, iatrogenic complication, foreign body injection, causes of medication errors, coring

## Abstract

Coring is the retention of material from a medication vial into the needle and syringe, which can ultimately be transfused into a patient, causing adverse outcomes. The purpose of this article is to increase awareness of this underreported finding and to propose solutions to improve the quality of care and decrease fatalities.

A 65-year-old male with a significant cardiovascular history was admitted and required an emergent bedside esophagogastroduodenoscopy, for which propofol was being aspirated for IV induction. This resulted in the coring of the vial topper and subsequently a rubber piece in the syringe.

The prevention of coring has largely focused on anticipating the shortcomings of currently available medication vials and aspiration techniques. However, these strategies have limitations. Further work can highlight risk-mitigating approaches such as different aspiration techniques, needle or vial types, and prefilled syringes. More importantly, these interventions may reduce perioperative morbidity and mortality.

## Introduction

Coring, the retention of material from a medication vial top inside the needle and syringe used to draw the medication, may occur depending on the vial stopper material, vial size, needle size and type, and the angle of puncture. As per a paper by Eskander et all., the incidence was quoted to vary widely between 3% and 97%, and the implications may include undetectable granuloma formation, localized ischemia where the cored material wedges after injection, anaphylactic reactions, organ failure, and death [1]. These outcomes are likely underreported. Our aim is to summarize the data and provide recommendations related to reducing such preventable morbidity.

## Case presentation

**Figure 1:**
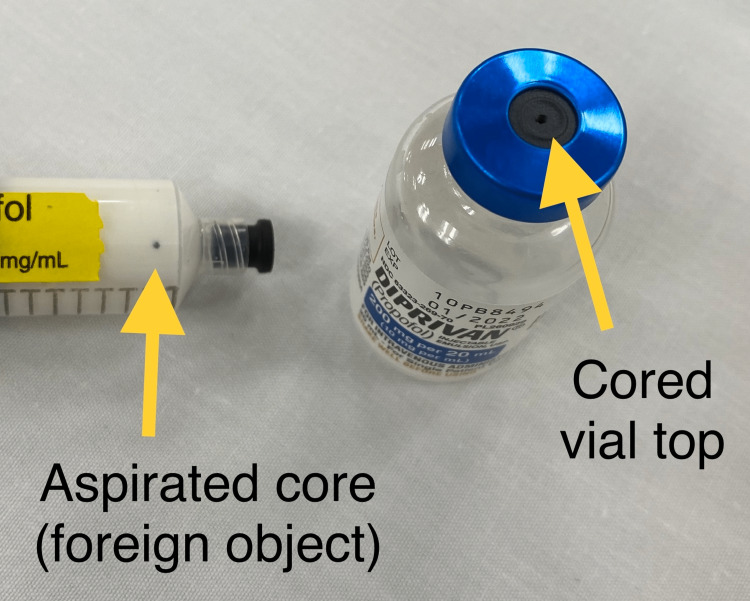
Rubber foreign body inside the propofol-labeled syringe This case report highlights a potential adverse event that could have occurred had the foreign body been injected into the patient’s circulatory system.

A 65-year-old American Society of Anesthesiologists (ASA) Physical Status 4E male with a history of coronary artery disease, type 2 diabetes mellitus, cerebrovascular events, and myasthenia gravis was admitted for a myasthenic crisis, acute-onset severe abdominal pain, and spurious hematochezia. The anesthesia team was consulted to provide anesthesia for an emergent bedside esophagogastroduodenoscopy due to a declining hemoglobin level (below 5 g/dL). During preparation for the case, a 20 mL vial of propofol (Fresenius-Kabi, Lake Zurich, IL, USA) was punctured and aspirated into a 20 mL syringe equipped with an 18 gauge 1 ½ inch blunt fill needle (BD Luer-Lok, Franklin Lakes, NJ, USA) as per usual practice. Upon aspiration, a foreign body was noted in the syringe (Figure 1). Upon closer examination, it was found that the foreign body was a cored piece of rubber from the medication vial stopper. The decision was made to waste the propofol vial, medication, syringe, and filling needle and draw up a new syringe for the case. The new syringe and its contents were examined carefully prior to injection and were not noted to contain any foreign material. Despite propofol being drawn up in preparation for the case, it was not utilized due to the hemodynamic instability of the patient. The patient underwent esophagogastroduodenoscopy with epinephrine injection and clip placement with successful hemostasis for an actively bleeding Dieulafoy lesion in the duodenal bulb. The procedure was performed without complications and was well tolerated.

## Discussion

Coring is easily missed due to difficulty in visualization because of its small size, the masking effect of the vial labels, or the medication opacity [2]. In reported cases, the consequences range from undetectable localized tissue trauma to severe morbidity and death [3]. Coring may only be detected with careful inspection of all medication syringes, after infusion pump tubing gets blocked, or when a patient exhibits signs of an adverse event [4]. This is a recognized and largely preventable source of patient harm and has mostly been addressed anecdotally in the published literature [2,3,5].

The prevention of coring has largely focused on encouraging manual inspection, changing the angle of entry into the rubber medication vial top, orienting the drawing needle bevel upward, controlling the drawing pressure, or changing the drawing needle and syringe size [6,7]. Some centers have elected to avoid the use of blunt drawing needles altogether [4], while others have advocated the use of specialized syringe attachments to decrease the incidence of coring [8], which has led to other complications related to those devices [9].

Many of these alterations in technique or needle specifications have their own demerits. For example, decreasing the needle size may reduce the rate of coring, but if coring does occur, the size of the foreign body will be reduced and may be difficult to detect in the syringe [6]. Decreasing needle size may also make it technically difficult to aspirate a large volume of medication into a syringe. Another suggested technique is inserting the needle into the stopper at a 45-degree angle with the bevel pointing upward, which may decrease the incidence of coring but will unlikely eliminate its occurrence. None of these techniques is fail-proof, and a coring event could potentially lead to significant morbidity despite adherence to suggested guidelines.

Rubber vial tops have been in medical use for a long time and are largely employed in multidose, large-volume, multiuse vials. This practice is currently being phased out in favor of using a single vial per patient to eliminate medication errors and source contamination. The result is an increased volume of medication waste and continued incidence of complications such as coring. Thus, the concept of a rubber top vial loses ground to more practical and less wasteful solutions, of which we propose prefilled syringes or other forms of single-use medication containers.

The salient features of this case stem from the urgency of the procedure (rapid blood loss from the Dieulafoy lesion), numerous patient comorbidities, and bedside location with limited resources and personnel. This case required rapid induction and careful hemodynamic monitoring in a patient with impending hemodynamic compromise from severe acute anemia. The coring event, if not recognized, could have led to serious adverse events. Regardless, even the recognized coring event consumed valuable time in a setting where seconds matter and rapid hemodynamic decompensation is a constant threat to patient outcomes. A reduction in the number of steps required to have the medication ready can potentially improve efficiency and safety for patients and minimize distractions for the providers.

Newer and safer practices of medication aspiration can be valuable for both providers and patients. Firstly, many patients who need surgical intervention may have comorbidities such as pulmonary disease, atherosclerotic disease, and bleeding diathesis, in which foreign body embolism can exacerbate the quality of life or cause death. Secondly, existing perioperative risks associated with surgery, such as atelectasis, pulmonary embolism, or pneumoniae combined with foreign body embolism, could worsen outcomes by causing serious decompensation and morbidity. Lastly, the quality and efficiency of patient care can be improved by eliminating the threat of coring since tasks can shift toward other essential functions.

## Conclusions

Coring is a potential cause of morbidity and mortality, especially in emergent situations, and is likely underreported. The currently proposed techniques to avoid coring include modifying the angle of injection, changing to small gauge needles, and anecdotally visualizing the syringe. To improve the standard of care and reduce morbidity and mortality, anecdotal studies suggest prefilled syringes, pre-attached syringe-vial combinations, and/or single-use medication containers. To better assist in this process, more research is needed to document the true incidence of coring among the traditional medication drawing practices and test which strategies of medication aspiration or infusion are most effective at decreasing coring rates.
